# Diphtheria

**Published:** 1890-07

**Authors:** Wm. Steinrauf

**Affiliations:** St. Charles, Mo.


### DIPHTHERIA.

WM. STEINRAUF, M. D., ST. CHARLES, MO.

There have been quite a few cases of diphtheria in our city during the past two months, and quite a number of deaths therefrom. In a good many cases the diphtheritic exudation was accompanied with what is known as scarlet rash or German measles. I will state right here that the use of high potencies has given the best results in these cases. One or two doses of the indicated remedy was generally, might say always, all-sufficient.

Nasal types of a severe form, such as we had them, were well met by Bromium, Lac-caninum, Lachesis, Lycopodium, and Sulphur. Most cases required Lachesis or Lycopodium. In the croupous forms Bromium or Lac-caninum; in the hæmorrhagic types Lachesis or Sulphur.

No swabs, no gargles, no external means of any kind were employed, and the results were all that could be desired.

When the last and greatest complication arises; no reaction, constantly and steadily sinking, cold and clammy skin, cold sweat, stupor, give Sulphur at once. These cases invariably die unless a dose or two of Sulphur is interposed.

Is there a prophylactic against diphtheria? For the last three years I have obtained Diphtherin$^{cm}$ (Swan), and in every epidemic since I have given my patients this remedy, two or three doses a week. With what results do you ask? So far I have never had a case of diphtheria where this was given as a preventive.—*American Homœopathist.*

